# Current Knowledge on the Vascular Effects of Menthol

**DOI:** 10.3389/fphys.2020.00298

**Published:** 2020-04-07

**Authors:** Henrique Silva

**Affiliations:** ^1^CBIOS - Universidade Lusófona’s Research Center for Biosciences and Health Technologies, Lisboa, Portugal; ^2^Pharmacol. Sc Depart - Universidade de Lisboa, Faculty of Pharmacy, Lisboa, Portugal; ^3^Department for Management of Science and Technology Development, Ton Duc Thang University, Ho Chi Minh City, Vietnam; ^4^Faculty of Pharmacy, Ton Duc Thang University, Ho Chi Minh City, Vietnam

**Keywords:** menthol, vascular, vasoconstriction, vasodilation, TRPM8 channels, calcium channels, review

## Abstract

Menthol is a monoterpene alcohol, widely used in several food and healthcare products for its particular odor and flavor. For some decades, menthol has been known to act on the vasculature directly in the endothelium and vascular smooth muscle, with recent studies showing that it also evokes an indirect vascular response via sensory fibers. The mechanisms underlying menthol’s vascular action are complex due to the diversity of cellular targets, to the interplay between signaling pathways and to the variability in terms of response. Menthol can evoke either a perfusion increase or decrease *in vivo* in different vascular territories, an observation that warrants a critical discussion. Menthol vascular actions *in vivo* seem to depend on whether the vascular territory under analysis has been directly provoked with menthol or is located deep/distant to the application site. Menthol increases perfusion of directly provoked skin regions due to a complex interplay of increased nitric oxide (NO), endothelium-derived hyperpolarization factors (EDHFs) and sensory nerve responses. In non-provoked vascular beds menthol decreases perfusion which might be attributed to heat-conservation sympathetically-mediated vasoconstriction, although an increase in tissue evaporative heat loss due the formulation ethanol may also play a role. There is increasing evidence that several of menthol’s cellular targets are involved in cardiovascular diseases, such as hypertension. Thus menthol and pharmacologically-similar drugs can play important preventive and therapeutic roles, which merits further investigation.

## General Characterization of Menthol

Menthol is a cyclic monoterpene alcohol present mainly in several species of Mentha herbs (mints), and is responsible for their distinctive cool/fresh odor and flavor (Eccles, 1994). The main source of menthol is peppermint, introduced in the European medical culture in the 18^th^ century. Peppermint essential oil has been used throughout the centuries for its therapeutic properties in a variety of medical disorders including gastrointestinal, respiratory, reproductive and inflammatory, among others (Lawrence, 2006). Menthol possesses particular odorant, flavoring and therapeutic properties, which justifies its use in many food and healthcare products, including cosmetics, dentifrices, analgesics, cough medicines, as well as in tobacco products (Craighead and Alexander, 2016).

**FIGURE 1:**
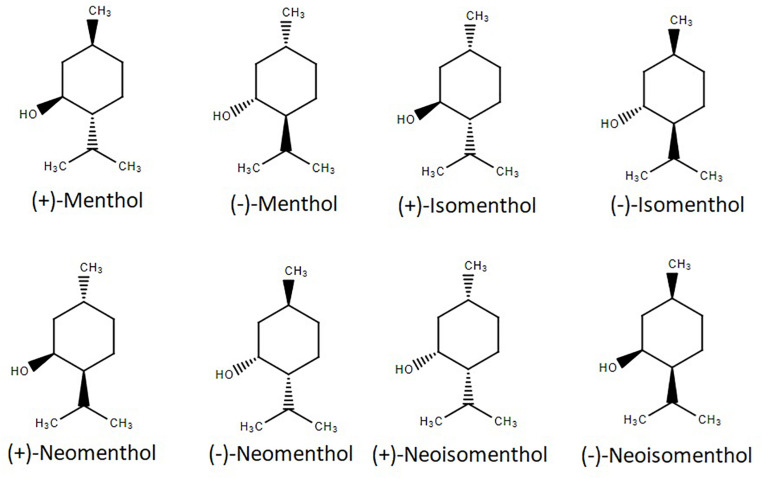
Structure of the four pairs of optical menthol isomers.

Menthol (5-methyl-2-propan-2-ylcyclohexan-1-ol) possesses three stereogenic centers and, therefore, four pairs of optical isomers: (+)- and (−)-menthol, (+)- and (−)-neomenthol, (+)- and (−)-neoisomenthol, and (+)- and (−)-isomenthol, whose structures are shown in Figure 1. Menthol has a molecular weight of 156.26 g/mol, is solid at room temperature (melting point: 41–44°C), is not completely soluble in water (431 mg/L at 20°C), but is a freely soluble ethanol (Kamatou et al., 2013). It has a log (octanol/water) value of 3.4, which means that it is a lipophilic molecule capable of interacting with biological membranes (Turina et al., 2006). Menthol was first isolated in a pure form in 1771 by Heidelberg-born chemist and physician Hieronymus David Gaubius (1705–1780). The first studies on its characterization and structure determination were published by Oppenheim, Beckett, Moriya and Atkinson in 1862–1882 (Schaefer, 2015). The only naturally-occurring enantiomer, isolated from a variety of mint herbs species, is (–)-menthol. The other optical isomer, (+)-menthol, is an industrial synthesis product that yields the racemic (±)-mixture (Oertling et al., 2007). (–)-menthol has a fresh, sweet, minty odor, while that produced by (+)-menthol is dusty, herbal and only fairly minty (Eccles et al., 1988). Furthermore, (+)-menthol pharmacological properties are vastly reduced compared to (−)-menthol (Eccles et al., 1988; Bhatia et al., 2008; Lawrence et al., 2011), and are reported as toxic (Petrowitz, 1980). Given its popularity, menthol is a highly demanded molecule, which justifies why most menthol today is not extracted from plants, but obtained by chemical synthesis (Schaefer, 2015).

Menthol absorbed via the gastrointestinal tract, skin or airways is transported to the liver by the circulation, where it is mainly metabolized. When taken orally, menthol undergoes marked rapid absorption in the small intestine (Hiki et al., 2011), whereas transdermal absorption is considerably slower and less extensive (Valente et al., 2015). Given its highly lipophilic nature, it is metabolized by uridine diphosphate-glucuronyltransferase to form menthol glucuronide (phase II reaction), which is then delivered to the kidneys for urinary excretion. This reaction is quick enough to maintain menthol at low (or undetectable) levels in venous blood, unlike the high levels detected of menthol glucuronide (Gelal et al., 1999; Hiki et al., 2011). A smaller fraction of menthol glucuronide is also excreted in feces, and undergoes intensive enterohepatic circulation. After the cleavage of glucuronide and reabsorption in the small intestine, it is further metabolized in the liver by cytochrome P450 2A6-mediated hydroxylation and oxidation reactions (phase I reactions). p-Menthane-3,8-diol and 3,8-dihydroxy-p-menthane-7-carboxylic acid have been identified as major metabolites in urine, and a fraction of these compounds is also excreted as glucuronides (Nakaoka, 1990; Miyazawa et al., 2011). Menthol metabolites are apparently pharmacologically inert, although menthol glucuronide has been investigated as a potential prodrug for oral administration (Nolen and Friend, 1994).

## Vascular Actions of Menthol

**FIGURE 2:**
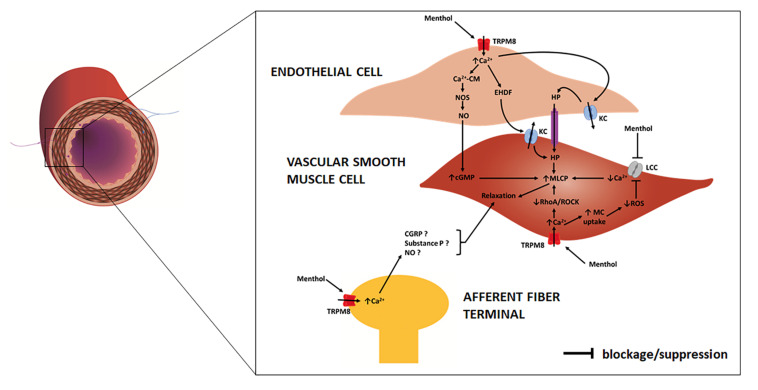
Schematic representation of an artery/arteriole depicting its intima, media and adventitial layers. Afferent (purple) and efferent (blue) nerve fibers are included. Highlight is given to the mechanisms responsible for menthol-induced vasodilation, as proposed in previous studies. In the endothelial cell menthol activates TRPM8 channels leading to extracellular calcium influx. Increased cytosolic calcium (1) binds to calmodulin (CM) with the resulting complex activating nitric oxide synthase (NOS) which increases NO release to VSM cells, where it increases cyclic guanosine monophosphate (cGMP); (2) induces the release of endothelium derived hyperpolarization factors (EDHFs) which opens potassium channels (KC) on VSM cells; (3) induces endothelial cell potassium channels opening leading to hyperpolarization (HP), which is communicated to VSM cells. In VSM cells menthol (4) blocks membrane L-type calcium channels (LCC), decreasing extracellular calcium influx; (5) activates membrane TRPM8 channels, leading to an increase in extracellular calcium influx, which suppresses RhoA/ROCK pathway. Increased cytosolic calcium triggers mitochondrial (MC) uptake, leading to a decrease in reactive oxygen species (ROS) production, which prevents opening of LCC. Increased cGMP, decreased cytosolic calcium concentration and suppressed RhoA/ROCK pathway contribute to an increase of myosin light chain phosphatase (MLCP) activity and VSM cell relaxation. On afferent A-delta and C fibers menthol may activate TRPM8 channels leading to an increased cytosolic calcium concentration and to the release of several substances, possibly calcitonin gene-related peptide (CGRP), substance P and NO, which lead to VSM cell relaxation.

**FIGURE 3:**
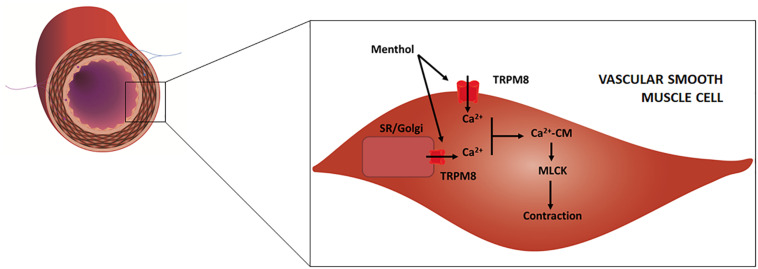
Schematic representation of an artery/arteriole depicting its intima, media and adventitial layers. Afferent (purple) and efferent (blue) nerve fibers are included. Highlight is given to the mechanisms responsible for menthol-induced vasoconstriction, as proposed in previous studies. Menthol activates TRPM8 channels located on (1) plasma membrane, leading to extracellular calcium influx, and (2) on the sarcoplasmic reticulum (SR) and/or Golgi apparatus leading to calcium release. Increased cytosolic calcium binds to calmodulin (CM) and the resulting complex activates myosin light chain kinase (MLCK) which leads to VSM contraction.

Historic records about the application of menthol-containing herbs or products as therapeutic agents for cardiovascular disorders are very scarce. Menthol pharmacological properties for the cardiovascular system only became apparent in the 20^th^ century with the first publications appearing in the 1920s. For some decades, menthol has been known to act on the vasculature directly in the endothelium or vascular smooth muscle (VSM), with recent studies showing that it also evokes an indirect vascular response via sensory fibers. The mechanisms underlying menthol’s vascular action are complex due to the diversity of cellular targets, to the interplay between signaling pathways and to the variability in terms of response. Menthol can evoke either a perfusion increase or decrease *in vivo* in different vascular territories, an observation that warrants a critical discussion. The next subsections present a comprehensive review of the main vascular actions attributed to menthol both *in vitro* and *in vivo* (animal models, human subjects), along with a critical discussion of the main *in vivo* findings to clarify these seemingly contradictory responses. Table 1 summarizes the findings of the most relevant *in vivo* studies to understand the actions of menthol on the vasculature. Figures 2,3 highlight the currently known mechanisms underlying both vasodilation and vasoconstriction responses observed with menthol application.

**TABLE 1:** Description and main results of the most relevant *in vivo* studies for characterizing the response of menthol in vasculature (y.o. – years old; m.o. – months old).

Author	Human/animal species and strain (sex)	Number and mean age of subjects	Measurement site	Type of application	Type of formulation	Menthol concentration	Perfusion measurement technique	Effect on perfusion
**Assessment of large caliber arteries**								
Olive et al. (2010)	Human, healthy subjects	*N* = 12; 24 y.o.	Arm	Topical	Gel	3.5%	High-resolution Doppler ultrasound	Brachial artery blood flow decrease
Topp et al. (2011a)	Human, healthy subjects	*N* = 17; 24 y.o.	Forearm	Topical	Gel	3.5%	High-resolution Doppler ultrasound	Radial artery blood flow decrease
Topp et al. (2011b)	Human, healthy subjects	*N* = 16; 24 y.o.	Thigh	Topical	Gel	3.5% and 10%	High-resolution Doppler ultrasound	Popliteal blood flow and caliber decrease
Topp et al. (2013)	Human, healthy subjects	*N* = 19; 26 y.o.	Forearm	Topical	Gel	3.5%	High-resolution Doppler ultrasound	Radial artery blood flow decrease
Hunter et al. (2018)	Human, healthy subjects (males)	*N* = 20; 21 y.o.	Thigh	Topical	Ethanolic solution	3%	High-resolution Doppler ultrasound	Femoral blood flow unchanged
Sun et al. (2014)	Humans, pre-hypertensive subjects	*N* = 18; 57 y.o.	Arm	Oral	Capsule	144 mg/day for 8 weeks	High-resolution ultrasound	Brachial artery blood flow increase
**Assessment of microcirculation – perfusion increase**								
Dölen et al. (2015)	Sprague-Dawley rats	*N* = 40; 3 m.o.	Skin flap	Direct	Gel	10%	Radionuclide scintigraphy	Blood flow increase
Hong and Shellock (1991)	Human, healthy subjects	*N* = 10; 34 y.o.	Foream	Topical	Eucalypmint	15%	Laser Doppler flowmetry	Blood flow increase
Wasner et al. (2004)	Human, healthy subjects	*N* = 10; 39 y.o.	Forearm	Topical	Ethanolic solution	40%	Laser Doppler flowmetry	Blood flow increase
Namer et al. (2005)	Human, healthy subjects	*N* = 10; 34 y.o.	Forearm	Topical	Ethanolic solution	40%	Laser Doppler imaging	Blood flow increase
Johnson et al. (2009)	Human, healthy subjects	*N* = 11; 23 y.o.	Forearm	Topical	Aqueous (72%) and ethanolic (25%) solution	3%	Laser Doppler flowmetry	Blood flow increase
Craighead and Alexander (2016)	Human, healthy subjects	*N* = 10; 24 y.o.	Forearm	Topical	Alcohol and water-based gel (Biofreeze^®^)	4%	Laser speckle contrast imaging	Blood flow increase
Hunter et al. (2018)	Human, healthy subjects (males)	*N* = 20; 21 y.o.	Thigh	Topical	Ethanolic solution	3%	Laser Doppler flowmetry	Blood flow increase
Craighead et al. (2017)	Human, healthy subjects (females)	*N* = 10; 23 y.o.	Forearm	Intradermal microdialysis	Lactated Ringer’s solution	0.1–500mM	Laser Doppler flowmetry	Blood flow increase
Craighead et al. (2017)	Human, healthy and subjects	*N* = 10; 50 y.o.	Forearm	Intradermal microdialysis	Lactated Ringer’s solution	0.1–500mM	Laser Doppler flowmetry	Blood flow increase
	Human, hypertensive subjects	*N* = 9; 53 y.o.						
**Assessment of microcirculation – perfusion decrease or unchanged**								
Yosipovitch et al. (1996)	Human, healthy subjects	*N* = 18; 47 y.o.	Forearm	Topical	Aqueous (10%) and ethanolic (80%) solution	10%	Laser Doppler flowmetry	Blood flow unchanged
Jason Gillis et al. (2015)	Human, healthy subjects	*N* = 16; 20 y.o.	Finger	Topical	Aqueous solution	0.05% and 0.2%	Laser Doppler flowmetry	Blood flow decrease
Valente et al. (2015)	Human, healthy subjects	*N* = 10; 24 y.o.	Hallux	Topical	Aqueous gel	10 mg/kg of body weight	Laser Doppler flowmetry	Blood flow decrease
		*N* = 10; 26 y.o.		Oral	Capsule			

### Direct Action on the Vascular Wall

#### Menthol Actions on TRPM8 Channels

The transient receptor potential melastatin-related 8 (TRPM8) channels are widely accepted to be the main targets of menthol in different cell types, including neurons and smooth muscle cells of several organs. TRPM8 channels are also implicated in the detection of cool/cold temperatures by afferent A-delta and C nerve fibers (Kobayashi et al., 2005) and menthol activation of these channels in skin and mucosae mimics these sensations. The vascular expression of TRPM8 channels seems to differ according to animal species and age, as well as to the type of vascular bed. For example, TRPM8 were identified in rat pudendal (Silva et al., 2015), aortae, tail and mesenteric arteries (Johnson et al., 2009), while another study failed to detect their presence in the mesenteric arteries (Inoue et al., 2006). TRPM8 channels have been detected in different cellular locations, namely the plasma membrane (Gai-ying et al., 2014) and in the membrane of intracellular calcium stores, namely Golgi apparatus (Mahieu et al., 2007) and sarcoplasmic reticulum (SR) (Eccles et al., 1988; Lawrence et al., 2011; Schaefer, 2015), although there are conflicting data regarding the latter (Mahieu et al., 2007).

In the vascular endothelium, extracellular calcium influx leads to calmodulin-mediated activation of nitric oxide synthase (NOS) which increases nitric oxide (NO) synthesis, and consequently leads to VSM relaxation and vasodilation (Cohen and Vanhoutte, 1995). Calcium also opens potassium channels, leading to hyperpolarization, which is communicated to VSM cells via gap junctions with consequent relaxation and vasodilation (Félétou and Vanhoutte, 2009). In VSM, menthol induces extracellular calcium influx together with calcium release from Golgi apparatus and/or SR, both responses being mediated by TRPM8 channels (Mahieu et al., 2007; Neumann and Copello, 2011; Melanaphy et al., 2012). However, SR-mediated calcium release can also be triggered independently of TRPM8 channels, although the mechanism remains unexplained (Mahieu et al., 2007). The rising cytosolic calcium concentration induces calmodulin-mediated myosin light chain kinase (MLCK) activation, which results in myosin light chain (MLC) phosphorylation and consequently in VSM contraction and vasoconstriction (Davis and Hill, 1999). This response has been demonstrated in endothelial-denuded vessels (Macpherson et al., 2006; Johnson et al., 2009; Silva et al., 2015), and observed in other smooth muscles, including the bladder (Jun et al., 2012), vas deferens (Boldyrev et al., 2009; Vladymyrova et al., 2011) and gastric fundus (Mustafa and Oriowo, 2005). It has also been proposed that TRPM8 channels might exist in cutaneous sympathetic vasodilator fibers, whereby released acetylcholine could lead to NO-dependent VSM relaxation (Johnson et al., 2009), although no studies have effectively investigated this hypothesis.

#### Menthol Actions on Voltage-Gated Calcium Channels

Menthol blocks L-type voltage-gated nifedipine-sensitive calcium channels in VSM cells of rat aortic, mesenteric, coronary (Cheang et al., 2013) and tail arteries (Melanaphy et al., 2016), among several other smooth muscles (Swandulla et al., 1987; Wright et al., 1997; Baylie et al., 2010; Cheang et al., 2013; Wang et al., 2016). This leads to a decrease in calcium intracellular concentration and consequently MLCK activity is overcome by that of myosin light chain phosphatase (MLCP) which leads to VSM relaxation and vasodilation.

#### Menthol Actions on RhoA/ROCK Pathway

There is also evidence to support that menthol causes vasodilation via suppression of the Rho-associated protein kinase (ROCK) pathway in VSM cells, presumably via TRPM8-dependent and TRPM8-independent mechanisms (Nunes et al., 2011; Xiong et al., 2017). The increase in cytosolic calcium concentration via L-type calcium channels and/or SR channels activates the RhoA/ROCK pathway. ROCK inactivates MLCP by phosphorylating its myosin-binding site, being unable to overcome MLCK activity, resulting in a sustained VSM contraction. By suppressing the RhoA/ROCK pathway, menthol increases MLCP activity, which is able to overcome MLCK activity, resulting in VSM relaxation (Fernández-Tenorio et al., 2011). Finally, menthol action on TRPM8 channels couples the release of calcium from SR stores to its uptake in the mitochondria. The increase in mitochondrial calcium uptake promotes the activity of pyruvate dehydrogenase and oxidative phosphorylation enzymes, which inhibit the production of ROS. This prevents further influx of extracellular calcium via L-type voltage-gated nifedipine-sensitive calcium channels (Xiong et al., 2017).

#### Menthol Actions on TRPA1 Channels

Transient receptor potential ankyrin transmembrane protein 1 (TRPA1) channels have been identified on endothelial cells of rat arteries (Earley et al., 2009; Pozsgai et al., 2010), more concentrated in the myoendothelial junction (Earley, 2012). Activation of endothelial TRPA1 channels leads to cell hyperpolarization which is communicated to VSM cells via gap junctions, with consequent VSM relaxation and vasodilation (Earley et al., 2009). Activation of VSM cell TRPA1 leads to RhoA/ROCK pathway activation and vasoconstriction (Aubdool et al., 2014). TRPA1 channels are also found in adventitial nerve fibers (Bautista et al., 2005). Afferent fibers TRPA1 channel activation detects local cooling together with TRPM8 channels (Pan et al., 2018) and can induce the release of CGRP, substance P and NO (Aubdool et al., 2014, 2016). Efferent sympathetic TRPA1 channel activation induces release of noradrenaline to VSM adrenergic receptors (Aubdool et al., 2014). Menthol actions on TRPA1 channels have striking differences according to species. While non-mammalian TRPA1 channels are insensitive to menthol (Xiao et al., 2008), mammalian TRPA1 respond differently depending on the concentration, showing a bimodal response. In mammalian cell cultures low (∼50–70 μM) menthol concentrations activate TRPA1 channels (Karashima et al., 2007), while high concentrations (≥250 mM) inhibit them (Macpherson et al., 2006; Karashima et al., 2007). In human TRPA1 channels, menthol only acts as an activator (Xiao et al., 2008). To date no studies reporting any action of menthol in vascular TRPA1 channels exist.

#### Menthol Actions on TRPV Channels

Transient receptor potential vanilloid-receptor-related (TRPV) channels 1–4 have been identified in murine and rat arteries and nerves (Earley and Brayden, 2015; Du et al., 2019). TRPV3 and TRPV4 are expressed both in endothelial and VSM cells, TRPV1 in endothelial cells and sensory nerve fibers, while TRPV2 were found only in VSM cells (Earley and Brayden, 2015). Activation of these receptors leads to vasodilation through NO-dependent, NO-independent, sensory-mediated mechanisms. To date, however, menthol has only been found to activate neuronal TRPV3 channels (Macpherson et al., 2006) and to block TRPV1 channels in cell cultures (Takaishi et al., 2016). Since there are no studies reporting a vascular action in these receptors, it is presently unclear whether any menthol vascular effect is attributed to its action on TRPV channels.

### Interaction of Ethanol on Menthol Receptors

Ethanol is commonly used as a solvent in several menthol-based formulations given the latter’s low water solubility. It is also known to interfere with the activity of both TRPM8 and TRPV1 channels. TRPM8 channel activation is dependent on the presence of membrane lipid phosphatidylinositol-4,5-biphosphate (PIP_2_) insofar as its removal by phospholipase enzyme isoforms contributes to this channel’s desensitization (Benedikt et al., 2007). Ethanol can diminish the TRPM8 response to menthol by weakening its interaction with PIP_2_ (Weil et al., 2005; Benedikt et al., 2007). Therefore, it is possible that ethanol-based menthol formulations may weaken menthol response both at the endothelium and VSM cells. TRPV1 channels are inhibited by PIP_2_, therefore ethanol can potentiate their response to menthol (Benedikt et al., 2007). Whether ethanol also interferes with the binding of menthol to TRPV1 channels is currently unknown.

Ethanol is itself a vasoactive substance, being able to induce either vasodilation or vasoconstriction in different vascular beds. Its best known effect is vascular relaxation, a response typically found in pulmonary (Greenberg et al., 1993), mesenteric (Ru et al., 2008), cutaneous (Altura and Altura, 1982, 1987) and cerebral blood vessels (Altura and Altura, 1999), which has been attributed to the potentiation of NO-dependent and NO-independent vasodilation pathways (Rocha et al., 2012; Tawakol et al., 2019). In certain vascular beds ethanol-induced vasodilation is rather attributed to its metabolite acetaldehyde, which mediates an endothelium-dependent relaxation (Jin et al., 2019) in mesenteric vessels. Furthermore, acetate, a metabolite of aldehyde also displays a moderate vasodilator response (Altura and Altura, 1982). In contrast, some vascular beds tend to be constricted by ethanol, such as the cerebral (Altura and Altura, 1982, 1987), coronary (Altura et al., 1983), mesenteric (Toda et al., 1983; Ru et al., 2008), aortic (Ru et al., 2008), an effect that may involve extra-endothelial TRPV1 channels (Cleland et al., 2019). Ethanol could interfere with the vascular action of menthol via direct and/or indirect mechanisms both *in vitro* and *in vivo*. It could be acting directly on the vascular wall, as well as indirectly by modulating the binding of menthol to TRPM8 and TRPV1 receptors. Either way, it is critical that experimental settings involving menthol administration in ethanol-based formulations address its intrinsic vascular effects (see Table 1).

### Menthol-Induced Vasoconstriction

In several *in vivo* studies carried out to date menthol has not been applied directly to the vessels via parenteral routes, but on the skin, either topically or via intradermal microdialysis. Perfusion has been assessed at the application site or at deeper/distant sites relative to the application site. In addition, a few studies have also explored the effects of orally administered menthol. An important observation has been made – large caliber arteries respond mainly to menthol application with perfusion decrease while cutaneous microcirculation responds mainly with perfusion increase.

Perfusion decrease has been consistently described in the brachial (Olive et al., 2010) and radial (Topp et al., 2011a, 2013) arteries after topical application on the forearm, and in the popliteal arteries (Topp et al., 2011b) after application on the thigh. In both cases, perfusion was assessed with high-resolution Doppler ultrasound, and decreased both ipsilateral and contralateral to the side of application (popliteal). Although this perfusion decrease is likely attributed to vascular caliber reduction, this was only observed in one study (Topp et al., 2011b), which can be attributed to the technique’s limited sensitivity (Topp et al., 2013). At first glance, these results suggest that menthol undergoes transdermal absorption, reaches systemic circulation and causes the constriction of these territories. In fact, one study reported that following application in dermal patches menthol reached systemic circulation in low but detectable levels (Martin et al., 2004). Although such low doses are unlikely to cause an observable effect, this cannot be discarded as the authors did not measure perfusion in areas far from the application site.

Another hypothesis is that perfusion decrease may be attributed to a decrease in tissue/organ temperature, not in response to menthol but rather to the ethanol contained in the formulation. In recent studies, topical menthol application caused a decrease in muscle (Hunter et al., 2018) and skin temperature (Lasanen et al., 2016), irrespective of concentration. In both cases this response was not attributed to a possible menthol action on the vasculature (not measured) but rather to an increase in evaporative heat loss caused by ethanol (Hunter et al., 2018), which may have directly caused vessel constriction. This again suggests that menthol permeation of muscle from topical application is not likely to occur. Again, it is also possible that ethanol interfered with TRPM8 channels, decreasing menthol activity, a hypothesis that needs further investigation.

A third hypothesis is that menthol may evoke neural-mediated thermoregulatory responses. It is possible that menthol action on TRPM8 and TRPA1 channels present in sensory A-delta and cold-sensory C fibers (Mahieu et al., 2007; Dhaka et al., 2008) can trigger a sympathetic-mediated heat-conservation constriction of large-caliber arteries (Pan et al., 2018). Evidence for the existence of menthol-evoked heat-conservation responses comes from studies reporting that menthol lowers temperature thresholds for virtually all the thermoregulatory responses to cooling (Kozyreva et al., 2010), which are attributed to TRPM8 channel activation (Camila Almeida et al., 2012). In humans, application of a menthol spray on the upper body results in a vasoconstriction response on finger microcirculation (Jason Gillis et al., 2015). Furthermore, whole-body application results in a slower decrease of rectal temperature comparing with cool water immersion, which was again attributed to peripheral vasoconstriction (Kounalakis et al., 2010) even though perfusion was not assessed. Similar results were found with the application of menthol on the trunk of mice (Tajino et al., 2019) and with the immersion of the upper extremities in cold water, in which menthol attenuated cold-induced restorative vasodilation (perfusion not assessed) (Kim and Lee, 2018). Taken together, these results suggest that application of menthol over a moderate/large skin surface initiates heat-conservation responses caused by cold perception. It is likely that cold perception could have resulted in the sympathetic-mediated decrease in the caliber of large blood vessels (Guimarães and Moura, 2001) deep to the application sites and on the microcirculation of non-provoked skin. This should be further investigated by assessing the caliber of the cutaneous branches of deep arteries during menthol application.

### Menthol-Induced Vasodilation

The microcirculation response to topically applied menthol seems to depend on whether the assessed skin region was directly provoked with menthol or not. In non-provoked skin menthol is reported to induce cutaneous temperature/perfusion decrease. Menthol gels (0.5, 4.6, 10%) cause skin temperature reduction irrespectively of concentration (Lasanen et al., 2016), which may be explained by an increase in evaporative heat loss induced by the formulation ethanol (10%). A similar explanation seems to match the observed decrease in skin microcirculatory perfusion after spraying menthol (0.05, 0.2%) on the torso and upper limbs (Jason Gillis et al., 2015). Similarly, the application of menthol (10 mg/kg of body weight) on the neck, arm and leg evokes perfusion reduction on the hallux (Valente et al., 2015). However, in this study perfusion was assessed during lower limb dependency (sitting), which is likely to have evoked the venoarteriolar reflex, further contributing to the observed perfusion decrease (Crandall et al., 2002).

In the majority of studies assessing provoked skin, menthol has been found to increase microcirculatory perfusion, either by topical application (Crandall et al., 2002; Wasner et al., 2004; Kobayashi et al., 2005; Topp et al., 2013; Craighead and Alexander, 2016; Kim and Lee, 2018) or intradermal microdialysis (Hong and Shellock, 1991; Guimarães and Moura, 2001). When topically applied, menthol passively diffuses through the epidermis and reaches the dermal microvascular networks. Even assuming that heat-conservation sympathetic vasoconstrictive reflex may occur in the provoked region, it is probably being offset by menthol-mediated vasodilation (Charkoudian and Clinic, 2010). To date only one study showed that topical application did not affect perfusion of the provoked region (Yosipovitch et al., 1996). In this study, menthol was applied in a highly concentrated (80%) ethanol solution, therefore raising the question whether ethanol-induced menthol evaporation may have occurred before any response could be measured. In addition, it is also reasonable to assume that the short application and recording periods may have thwarted the observation of any vascular response. When administered *via* intradermal microdialysis, menthol is directly delivered to the dermal microvascular networks, therefore reducing the contribution of heat-conservation responses initiated by cold perception in the epidermal sensory fibers. Studies that investigated the vascular response with this technique also benefited from the use of lactated Ringer’s solution as a solvent instead of ethanol (Craighead and Alexander, 2017; Craighead et al., 2017). In these conditions, menthol consistently induced perfusion increase, which is dose-dependent and results from the interplay of sensory, NO-dependent and NO-independent (EDHFs) mechanisms (Craighead et al., 2017).

Currently, several mechanisms are known to contribute to menthol-induced vasodilation: (1) endothelial TRPM8 activation leading to an increase in NO release (endothelial NO-dependent response); (2) VSM L-type voltage-gated calcium channel blockade; (3) RhoA/ROCK pathway suppression. Several other mechanisms have been proposed to contribute to this response, although they need to be experimentally tested. For example, menthol co-activation of TRPA1 and TRPV channels in sensory afferent fibers, endothelial and/or VSM cells have been proposed (Craighead et al., 2017), even though no studies have reported that menthol binds these receptors in vascular cells. Menthol may induce vasodilation via generation of heat-gain responses that more specifically target brown adipose tissue and perivascular adipose tissue, which are sources of heat and adipose-derived relaxing factors, respectively (Gollasch, 2012). These tissues are known to express TRPM8 channels and to initiate thermogenesis in response to cold and menthol activation (Ma et al., 2012). Interestingly, recent studies report that increased thermogenic activity of perivascular adipose tissue has been associated with improved endothelial function and protection from vascular disease (Chang et al., 2012, 2013).

### Anti-Hypertensive Effects of Menthol

Parenteral and oral routes have also suggested a considerable BP lowering capacity for menthol. The anti-hypertensive potential of menthol was first recognized by Rakieten in 1957 with the intravenous administration to lower BP in cats and rabbits (Rakieten, 1957). When administered orally, the effects of menthol are dependent on the dose and duration of exposure. In healthy humans, a single oral administration of 100 mg of menthol is reported not to influence BP (Gelal et al., 1999) and therefore does not seem to evoke a vascular response. A higher dose (10 mg/kg of body weight), however, does evoke a cutaneous perfusion reduction (BP not measured) (Valente et al., 2015). In contrast, prolonged oral supplementation lowers blood pressure in hypertensive rodents (Sun et al., 2014; Xiong et al., 2017) and in pre-hypertensive humans. In the latter it also increases flow-mediated vasodilation (Sun et al., 2014), suggesting an improvement in endothelial function (Roustit et al., 2016). These effects are partly explained by the suppression of the RhoA/ROCK pathway by menthol, which was found to increase in human (primary and environmentally-induced) and experimentally-induced hypertension (Nunes et al., 2011; Xiong et al., 2017). Several other cellular targets of menthol are also implicated in hypertension. For example, a recent study suggests that angiotensin II induces VSM TRPM8 channel down-regulation in renovascular hypertension (Huang et al., 2017) and in pulmonary hypertension (Liu et al., 2013). Therefore, it is logical that the activation of TRPM8 channels by menthol can also contribute to this blood pressure lowering response.

## Conclusion

Menthol is a versatile molecule with complex actions on the vasculature which remain far from clear. Several cellular targets have been identified in both endothelial and VSM cells as well as in afferent nerve fibers involving a crosstalk between multiple signaling pathways. Menthol is known to activate TRPM8 channels in the endothelium, VSM and vascular afferent nerve fibers, while also blocking VSM L-type calcium channels. Several other menthol targets have been identified in non-vascular tissues including TRPA1, TRPV1 and TRPV3 channels, but whether menthol also acts on vasculature via these channels is still undetermined. Menthol vascular action *in vivo* seems to depend on whether the vascular territory under analysis has been directly provoked with menthol or is located deep/distant to the application site. Menthol increases perfusion of directly provoked skin regions due to a complex interplay of increased NO, EDHFs and sensory nerve responses. In non-provoked vascular beds menthol induces vasoconstriction which might be attributed to heat-conservation sympathetically-mediated vasoconstriction, although an increase in tissue evaporative heat loss due the formulation ethanol may also play a role. There is increasing evidence that several of menthol’s cellular targets are involved in cardiovascular diseases, such as hypertension. Thus menthol and pharmacologically-similar drugs can play important preventive and therapeutic roles, which merits further investigation.

## Author Contributions

HS contributed literature research and manuscript editing.

## Conflict of Interest

The author declares that the research was conducted in the absence of any commercial or financial relationships that could be construed as a potential conflict of interest.

## Abbreviations

BP: blood pressure
CGRP: calcitonin gene-related peptide
CM: calmodulin
EDHFs: endothelium-derived hyperpolarization factors
MLC: myosin light chain
MLCP: myosin light chain phosphatase
MLCK: myosin light chain kinase
NO: nitric oxide
NOS: nitric oxide synthase
PIP_2_: phosphatidylinositol-4,5-biphosphate
ROCK: Rho-associated protein kinase
ROS: reactive oxygen species
SR: sarcoplasmic reticulum
VSM: vascular smooth muscle.
