# Corrigendum: Current Knowledge on the Vascular Effects of Menthol

**DOI:** 10.3389/fphys.2020.602231

**Published:** 2020-10-20

**Authors:** Henrique Silva

**Affiliations:** ^1^CBIOS — Universidade Lusófona's Research Center for Biosciences and Health Technologies, Lisboa, Portugal; ^2^Pharmacol. Sc Depart – Universidade de Lisboa, Faculty of Pharmacy, Lisboa, Portugal; ^3^Department for Management of Science and Technology Development, Ton Duc Thang University, Ho Chi Minh City, Vietnam; ^4^Faculty of Pharmacy, Ton Duc Thang University, Ho Chi Minh City, Vietnam

**Keywords:** menthol, vascular, vasoconstriction, vasodilation, TRPM8 channels, calcium channels, review

In the published article, there was an error regarding the author's affiliations. As well as having affiliations 3 and 4, they should also have CBIOS — Universidade Lusófona's Research Center for Biosciences and Health Technologies, Lisboa, Portugal and Pharmacol. Sc Depart – Universidade de Lisboa, Faculty of Pharmacy, Lisboa, Portugal.

The author apologizes for this error and states that this does not change the scientific conclusions of the article in any way. The original article has been updated.

